# The Physicians Visiting List for 1885

**Published:** 1884-12

**Authors:** 


The Physician's Visiting List for 1885.—Thirty Fourth year of its publications, P. Blakiston, Son & Co., Philadelphia. The present list contains the same valuable information which has distinguished this acceptable publication for so many years, and its arrangement makes it a useful engagement book for the dental as well as the Medical Practitioner. Its use for a long period confirms its value, and its size and style make it very convenient for the purposes for which it is designed by its publishers.

